# Genome Sequences for Levilactobacillus brevis Autochthonous to Commercial Cucumber Fermentations

**DOI:** 10.1128/mra.00029-22

**Published:** 2022-04-12

**Authors:** Clinton A. Page, Ruby A. Carter-Ogden, Alice M. Lee, Ilenys M. Pérez-Díaz

**Affiliations:** a USDA-Agricultural Research Service, Food Science and Market Quality and Handling Research Unit, Raleigh, North Carolina, USA; b North Carolina State University, Department of Biological Sciences, Raleigh, North Carolina, USA; Montana State University

## Abstract

We report the whole-genome sequences, along with annotations, of 11 Levilactobacillus brevis isolates from commercial cucumber fermentations performed in North Carolina (*n* = 9) and Minnesota (*n* = 2), USA.

## ANNOUNCEMENT

Levilactobacillus brevis is a lactic acid bacterium of relevance in the production of fermented foods, including kefir, pickled vegetables, and sauerkraut, and is also a common cause of spoilage in beer making (1). L. brevis may be of use in the development of starter cultures for pickling to maintain microbial stability due to its ability to utilize xylose and l-citrulline, which might otherwise provide an energy source for the spoilage-associated organism Lentilactobacillus buchneri (2).

We present 11 genome sequences and annotations corresponding to L. brevis strains isolated from commercial cucumber fermentations conducted in 2009 and 2010 in North Carolina and Minnesota, respectively (3). The L. brevis isolates were collected on day 3, 7, or 30 of the commercial fermentations at a collection tank depth of 2 or 8 feet from the brine surface. Isolation day and depth are incorporated into the genome sequence identification number. For example, genome sequence 3.8.25 was generated from a single colonial isolate (number 25) plated from a fermentation brine sample collected on day 3 at a depth of 8 feet.

All isolates were obtained from cucumber fermentation brines that had been spiral plated on *Lactobacillus* de Man-Rogosa-Sharpe (MRS) agar supplemented with 0.0001% cycloheximide solution and incubated at 30°C (3). Anaerobic growth conditions were maintained via the GasPak EZ system (BD, Franklin Lakes, NJ), which maintains an anaerobic atmosphere with ≥10% carbon dioxide. Isolated colonies were streaked on MRS agar prior to preparation of frozen stocks in MRS broth supplemented with 1.5% glycerol. Pure cultures were transferred to MRS broth from frozen stocks prior to DNA extraction. Cultures were incubated statically at 30°C. DNA extraction was conducted using the Wizard high-molecular-weight (HMW) extraction kit (Promega, Madison, WI). Bacterial isolates were preliminarily identified using the partial sequence of the 16S rRNA gene as described by Pérez-Díaz et al. (3). CosmosID (Rockville, MD) prepared libraries for Illumina reads with the Illumina Nextera XT kit and assessed libraries for quality with a Qubit fluorometer (Thermo Fisher Scientific) prior to whole-genome sequencing. Samples were sequenced on a NextSeq 550 platform (Illumina, San Diego, CA), producing paired-end reads with a maximum length of 150 bases. Raw sequence data were trimmed for adapters and low-quality bases using BBDuk (https://sourceforge.net/projects/bbmap), applying standard parameters (Phred quality, trimq=22; minimum length, minlen=36).

The initial assembly and annotation were performed in PATRIC (4). *De novo* assemblies were performed with Unicycler version 0.4.8 (5) with a minimum contig cutoff value of 300 bp. Quality assessment of assemblies was performed with QUAST version 5.0.2 (6), SAMtools version 13 (7), and Pilon version 1.23 (8). Assembled genomes were annotated in RASTtk (9). The closest reference genomes were identified by Mash/MinHash employing the PATRIC database (10). Upon submission to GenBank, assemblies were reannotated using the NCBI Prokaryotic Genome Annotation Pipeline (PGAP) (11). PGAP identified all isolates as belonging to L. brevis. Default parameters for the software were used.

### Data availability.

Assemblies were submitted to GenBank under BioProject accession number PRJNA674638. Strain identification and accession numbers for the genome annotations and Sequence Read Archive (SRA) data are included in Table 1.

**TABLE 1:** Accession numbers and genome statistics for 11 Levilactobacillus brevis isolates

L. brevis strain	GenBank accession no.	SRA accession no.	Assembly size (bp)	No. of contigs	Estimated coverage (×)	Total no. of reads	GC content (%)	*N*_50_ (bp)	Site of sample collection^*a*^
3.8.25	JAJHNO000000000	SRR16612720	2,503,685	173	376.39	3,542,171	45.65	40,343	NC
7.2.12	JAJHOC000000000	SRR16675615	2,559,005	199	528.447	4,814,899	45.45	40,344	NC
7.2.40	JAJHVM000000000	SRR16675614	2,557,347	178	423.63	3,908,850	45.48	40,343	NC
7.2.41	JAJHVN000000000	SRR16675613	2,452,927	168	989.898	9,553,893	45.75	37,182	NC
7.2.49	JAJHVO000000000	SRR16675612	2,497,582	159	148.893	1,488,733	45.65	40,343	NC
7.2.13	JAJHVP000000000	SRR16675611	2,542,145	213	449.91	4,265,009	45.57	36,178	NC
7.8.33	JAJHVQ000000000	SRR16675610	2,493,341	161	246.631	2,344,517	45.66	40,343	NC
7.8.34	JAJHVR000000000	SRR16675609	2,470,436	177	580.085	5,251,935	45.69	37,182	NC
7.8.43	JAJHVS000000000	SRR16675608	2,473,125	174	469.725	4,297,590	45.68	39,858	NC
30.2.29	JAJHVT000000000	SRR16675605	2,593,201	211	936.504	9,012,759	45.48	35,670	MN
30.8.38	JAJHVU000000000	SRR16675616	2,535,652	192	157.08	1,667,880	45.59	39,859	MN

a NC, North Carolina, USA; MN, Minnesota, USA.
